# Associations between home death, demographic factors and health care service usage for people who died in Scotland before and during the Covid-19 pandemic in Scotland

**DOI:** 10.23889/ijpds.v8i2.2220

**Published:** 2023-09-14

**Authors:** Jan Savinc

**Affiliations:** 1 United Kingdom, Scottish Centre for Administrative Data Research, Edinburgh, United Kingdom

## Objectives

To compare pre-pandemic and pandemic associations between death at home and: 1) demographic characteristics, 2) health care usage characteristics of people who died in Scotland.

## Method

Retrospective observational study of linked routine data: death registrations of all adults who died aged 18+ in Scotland during 12-month pandemic period (2020-03-23 to 2021-03-22) and pre-pandemic period (5 years prior), linked to inpatient acute hospital, psychiatric, and cancer registration records, and unscheduled care records (NHS24 calls, Accident & Emergency, ambulance, and Out-of-hours primary care records). Service usage will be summarised for pandemic and pre-pandemic cohorts. Separate logistic regression models for the pre-pandemic and pandemic periods will be used to test associations between demographic and service usage data, and death at home.

## Results

Deaths at home increased by about 35% during the first year of the pandemic in Scotland. N=285,770 people died in the 5 years before the pandemic and N=65,375 people died during the pandemic, of which N=76,770 and N=21,705 died at home, respectively. The associations between health service usage, demographic characteristics, and death at home changed during the pandemic.

## Conclusion

Studying the effects of the pandemic on end-of-life care has important implications for policy and future pandemic preparedness. Understanding how factors associated with home death changed during the pandemic will help inform care delivery for the increased number of deaths happening at home in Scotland.

